# The Impact of Remote Patient Monitoring on Clinical Outcomes in Heart Failure Patients: A Meta-Analysis

**DOI:** 10.7759/cureus.92812

**Published:** 2025-09-20

**Authors:** Kenneth Ezimoha, Mustafa Faraj, Sneha Kanduri Hanumantharayudu, Pavan Kumar Makam Surendraiah, Muhammad Zaigham Hassan, Abdulgafar D Ibrahim, Mamoun Jaber, Shivani Shah, Mounira Mefti, Gyullu Niftalieva, Bubli Ahmed, UFN Rizwanullah

**Affiliations:** 1 Medicine, The Grand Rehabilitation and Nursing at Queens, New York, USA; 2 Clinical Research, Wayne State University/Detroit Medical Center, Detroit, USA; 3 Medicine, The Partners Care, New York, USA; 4 Medicine, Westchester Medical Center, New York, USA; 5 Nephrology, Harlem Hospital Center, New York, USA; 6 Internal Medicine, Mississippi Baptist Medical Center, Jackson, USA; 7 Cardiology, Henry Ford Hospital, Detroit, USA; 8 Medicine, Caribbean Medical University School of Medicine, Willemstad, CUW; 9 Internal Medicine, Université Mouloud Mammeri de Tizi-Ouzou, Tizi Ouzou, DZA; 10 Medicine, Donetsk National Medical University, Donetsk, UKR; 11 Obstetrics and Gynecology, Jalalabad Ragib-Rabeya Medical College and Hospital, Sylhet, BGD; 12 Internal Medicine, Hayatabad Medical Complex Peshawar, Peshawar, PAK

**Keywords:** heart failure, hospitalization, meta-analysis, mortality, quality of life, remote monitoring

## Abstract

Remote patient monitoring (RPM) is increasingly used in heart failure (HF) management, but its clinical impact varies across studies. This systematic review and meta-analysis included 15 primary studies (nine randomized controlled trials and six observational cohorts) evaluating RPM effects on hospitalizations, mortality, and quality of life (QoL). In pooled analyses, RPM reduced HF-related hospitalizations (risk ratio (RR) = 0.80, 95% CI: 0.77-0.84, p < 0.0001). Implantable hemodynamic monitoring devices (e.g., pulmonary artery pressure sensors and cardiac implantable electronic devices) showed larger effects (RR = 0.72, 95% CI: 0.70-0.75) compared with noninvasive RPM modalities (e.g., telemonitoring, mobile apps; RR = 0.83, 95% CI: 0.81-0.86; p < 0.0001 for subgroup interaction). Mortality reduction was small but statistically significant (RR = 0.92, 95% CI: 0.90-0.94, p < 0.05), with implantable devices slightly stronger (RR = 0.90, 95% CI: 0.87-0.93) than noninvasive modalities (RR = 0.93, 95% CI: 0.91-0.96; p = 0.0074 for subgroup interaction). QoL showed a small, consistent improvement (standardized mean difference = 0.23, 95% CI: 0.20-0.26, p < 0.05) across MLHFQ and KCCQ instruments. Funnel plots and Egger’s regression indicated no publication bias for hospitalization (p = 0.45) or mortality (p = 0.62). Heterogeneity was low (I² = 0%, τ² = 0), with narrow prediction intervals. GRADE certainty was moderate for hospitalization and QoL and low for mortality. RPM, particularly implantable monitoring, reduces HF hospitalizations and improves QoL, with modest mortality benefits needing further confirmation. Implementation should target high-risk patients (NYHA III-IV and recent hospitalization), with future research on long-term survival, cost-effectiveness, and equitable access.

## Introduction and background

Heart failure (HF) is a progressive clinical syndrome caused by structural or functional cardiac abnormalities that impair the ability of the ventricles to fill or eject blood [1,2]. It affects over 64 million people worldwide and remains a leading cause of hospitalization and mortality [3,4]. With an aging population and advances in acute cardiovascular treatment, the prevalence of chronic HF continues to rise, creating an urgent need for strategies that can improve outcomes and reduce healthcare burden [5,6].

Remote patient monitoring (RPM) has emerged as a promising approach in HF management. RPM refers to the use of digital technologies to collect patient data in real time and transmit it to healthcare providers for timely review and intervention [7]. For clarity, RPM modalities can be grouped into four categories: (1) implantable pulmonary artery pressure sensors (e.g., CardioMEMS); (2) remote diagnostics from cardiac implantable electronic devices such as implantable cardioverter-defibrillators/cardiac resynchronization therapy devices; (3) noninvasive telemonitoring of vital signs (e.g., weight, blood pressure, heart rate, and oxygen saturation); and (4) mobile health apps and symptom-based check-ins. In contrast, structured telephone support, teleconsultation, and telerehabilitation are not considered RPM for this review.

RPM aims to enable earlier detection of clinical deterioration, reduce hospital readmissions, and promote patient self-management [8]. However, outcomes vary depending on patient population (ambulatory vs. post-discharge and HFrEF vs. HFpEF) and the type and intensity of monitoring. For example, the CHAMPION trial of implantable hemodynamic monitoring showed reduced HF hospitalizations [9], whereas the Tele-HF and BEAT-HF trials of post-discharge telemonitoring found no benefit [10,11]. More recently, TIM-HF2 [12] demonstrated a reduction in hospital days, while the large GUIDE-HF trial was neutral overall but suggested benefit pre-COVID [13]. These contrasting findings highlight the importance of distinguishing modalities, settings, and populations.

Several systematic reviews have synthesized the RPM evidence base [14-16]. Yet, many are now outdated, lack stratification by modality, or do not apply design-appropriate bias assessment (RoB 2 for randomized trials and ROBINS-I for cohorts). Meanwhile, digital health tools have advanced rapidly, and new large-scale randomized controlled trials (RCTs) and meta-analyses have been published since 2020. A contemporary synthesis is needed to clarify the effectiveness of RPM in HF management.

Guidelines reflect this uncertainty. The 2022 American Heart Association/American College of Cardiology/Heart Failure Society of America guideline recommends pulmonary artery pressure monitoring for selected high-risk patients (e.g., NYHA class III with prior hospitalization) but does not endorse broad use of RPM across all HF populations [17]. European guidance similarly supports the selective use of implantable sensors while regarding other RPM modalities as investigational.

This systematic review and meta-analysis evaluate the effect of RPM on key clinical outcomes in HF. The primary outcome is HF-related hospitalization. Secondary outcomes include all-cause mortality, health-related quality of life (QoL), and ED visits. We also prespecified subgroup analyses by modality (implantable vs. noninvasive), study design (RCT vs. cohort), and follow-up duration. We aim to provide a balanced synthesis of the evidence using contemporary trials, appropriate risk-of-bias tools, and GRADE certainty ratings to guide clinicians and policymakers on the role of RPM in HF care.

## Review

Methodology

Search Strategy and Study Selection

This systematic review was conducted in accordance with the Preferred Reporting Items for Systematic reviews and Meta-Analyses (PRISMA) 2020 guidelines. No PROSPERO registration was undertaken because the database searches had already commenced by the time the protocol was finalized, making registration ineligible. Nevertheless, all methods were prespecified in advance and fully documented to ensure transparency and reproducibility. A comprehensive search was carried out in PubMed, Embase, Scopus, Web of Science, and Cochrane CENTRAL from database inception to July 31, 2025. The strategy combined MeSH and free-text terms for heart failure, remote patient monitoring, telemonitoring, digital health, and specific devices such as CardioMEMS. Boolean operators, truncation, and filters were applied for human adults. Searches were conducted in PubMed, Embase, Scopus, Web of Science, and Cochrane CENTRAL using combinations of terms for heart failure (HF, HFrEF, and HFpEF) and remote monitoring (RPM, telemonitor, digital health, mHealth, eHealth, wearable, and CardioMEMS). Additional sources included ClinicalTrials.gov, the WHO International Clinical Trials Registry Platform (ICTRP), and gray literature. Reference lists of included trials and relevant reviews were also hand-searched.

All identified records were exported to EndNote X9, deduplicated, and screened in Rayyan QCRI by two independent reviewers. Titles and abstracts were assessed against eligibility criteria, and potentially relevant full texts were retrieved. Disagreements were resolved by consensus or a third reviewer. Screening reliability was quantified using Cohen’s κ. A PRISMA flow diagram presents the numbers identified, screened, excluded, and included.

Inclusion Criteria

Studies were deemed to be included in case they contained both adults with an HF diagnosis of age 18 years and above, terminal or ejection fraction (HFrEF), and terminal or fixed ejection fraction (HFpEF). The intervention was required to be some sort of RPM, which could include telemonitoring, mobile health applications, wearable sensors, and implantable devices, and be compared with another standard care model that lacked remote monitoring. Studies were also expected to report at least one pertinent clinical outcome, such as all-cause mortality, HF-related hospitalizations, ED visits, or QoL. They included only RCTs and controlled observational studies (cohort or case-control designs) published in English.

Exclusion Criteria

Studies were excluded from the analysis if they focused solely on device testing or technical development without reporting clinical outcomes. Additionally, any studies that were classified as case reports, editorials, conference abstracts, or reviews were also excluded. Furthermore, studies that included pediatric populations or populations not related to HF were not considered. Finally, any research that did not involve RPM was excluded from this meta-analysis.

Data Extraction and Quality Assessment

Two reviewers independently extracted data using a standardized form. Information included: study design, country, sample size, patient characteristics (age, sex, NYHA class, and HF phenotype), intervention type and duration, comparator, follow-up, and outcomes. Effect measures (risk ratios (RRs), HRs, and ORs) with 95% CIs were extracted; adjusted estimates were prioritized. For QoL, mean differences or standardized mean differences (SMD, Hedges’ g) were retrieved alongside the instrument used (e.g., KCCQ and MLHFQ).

Disagreements were resolved by consensus or a third reviewer. Missing or unclear data were clarified through supplementary material or author contact, where feasible.

Risk of bias was assessed separately for each study design. RCTs were evaluated with the Cochrane Risk of Bias 2 (RoB 2) tool across domains of randomization, deviations from intended interventions, missing data, outcome measurement, and reporting. Observational cohorts were appraised using ROBINS-I, covering confounding, participant selection, classification, deviation from interventions, missing data, measurement, and reporting. Judgments were categorized as Low, Some concerns, or High (RoB 2), and Low, Moderate, Serious, or Critical (ROBINS-I).

Two reviewers conducted risk-of-bias assessments independently. Results were visualized using traffic-light plots and tabulated summaries. Inter-rater reliability for screening was quantified with Cohen’s κ. Overall certainty of evidence was evaluated with the GRADE framework, considering risk of bias, inconsistency, indirectness, imprecision, and publication bias, with evidence rated as high, moderate, low, or very low certainty [17,18].

Statistical Analysis

Meta-analyses were performed using R (meta and metafor packages). For dichotomous outcomes (hospitalization and mortality), pooled RRs with 95% CIs were calculated. Time-to-event outcomes were analyzed as log-HRs with the generic inverse-variance method. For QoL outcomes, SMDs (Hedges’ g) were pooled.

A random-effects model with restricted maximum likelihood (REML) and Hartung-Knapp adjustment was applied. Between-study heterogeneity was quantified with I², τ², and 95% prediction intervals. Zero-event studies were handled using continuity corrections (treatment-arm continuity or Sweeting’s method).

Prespecified subgroup analyses included intervention modality (implantable vs. noninvasive), study design (RCT vs. cohort), and follow-up duration (<6, 6-12, and >12 months). Sensitivity analyses tested robustness by excluding high-risk-of-bias studies and small studies (n < 200) and comparing REML vs. DerSimonian-Laird.

Publication bias was assessed by funnel plots and Egger’s regression, restricted to outcomes with ≥10 studies. All analyses used a significance threshold of p < 0.05.

Results

Study Selection and Characteristics

**Figure 1:**
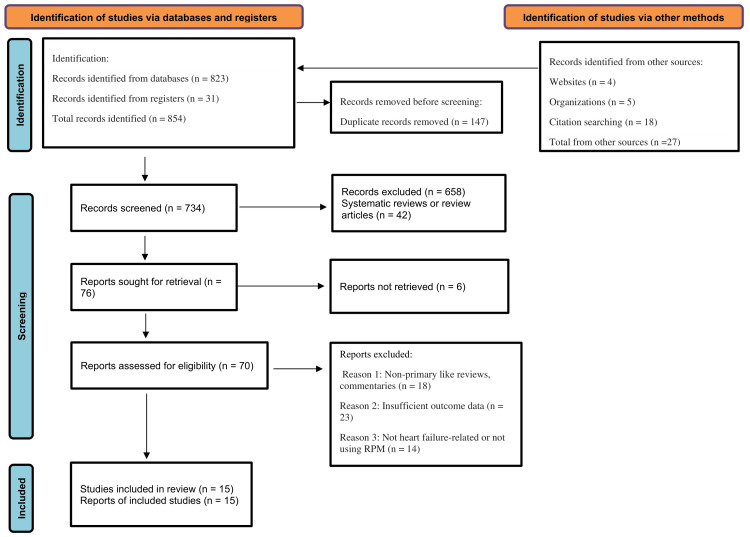
PRISMA flow chart for study selection PRISMA, Preferred Reporting Items for Systematic reviews and Meta-Analyses

After a thorough literature search using five renowned databases, including PubMed, Embase, Scopus, Web of Science, and Cochrane CENTRAL, 854 distinct records were identified. Following an elimination of 147 duplicates, 734 records would be screened. The screening of the title and abstract excluded 658 articles, with 42 of them systematic or narrative reviews. Seventy-six full-text articles comprising the possibly eligible studies were downloaded and evaluated. Among them, six reports could not be found, and 55 were not included due to reasons that include unavailability of primary outcome data, non-HF focus, and non-inclusion of an RPM aspect. Finally, it was found that 15 studies fulfilled the inclusion criteria to be included in the meta-analysis and systematic review (Figure 1).

The nine RCTs and six observational cohort studies were included and published from 2016 to 2025. The sample sizes were between 120 and 1,450 persons. Various RPM methods were used in the different interventions that were telemonitoring (n = 7), wearable sensors (n = 4), mobile health apps (n = 2), and implantable pulmonary artery pressure monitors (n = 2). The net follow-ups were between three months and 24 months, and the main outcome measures were death, admission to hospital, and QoL.

Studies Characteristics 

Table 1 provides a summary of the characteristics of the 15 included studies. Each study reported on patients diagnosed with HF, with most enrolling participants with NYHA class II-IV. Nine studies were multicenter RCTs, while the remaining six were prospective observational cohort studies. The remote monitoring interventions varied, with the most common being telemonitoring of vital signs, followed by smartphone-based symptom tracking and implantable monitoring systems. Comparator groups in all studies received usual care. Outcomes assessed included mortality, HF-related hospitalization, ED visits, and changes in health-related QoL. The diversity in intervention modalities and outcome measurement tools reflects the evolving landscape of RPM in the management of clinical HF.

**Table 1:** Characteristics of included studies CIED, cardiac implantable electronic device; HF, heart failure; mHealth, mobile health; NYHA, New York Heart Association; PA, pulmonary artery; QoL, quality of life; RCT, randomized controlled trial; RPM, remote patient monitoring

Study	Study design	Sample size	NYHA class	Intervention type	Comparator	Outcomes assessed	Follow-up duration
Koehler et al. [19]	Multicenter RCT	1,450	II-IV	Telemonitoring (vital signs)	Usual care	Mortality, HF-related hospitalization, and QoL	12 months
Abraham et al. [20]	Multicenter RCT	550	III-IV	Implantable PA pressure sensor (CardioMEMS)	Usual care	HF-related hospitalization and mortality	15 months
Ong et al. [21]	Multicenter RCT	1,437	II-IV	Telemonitoring (weight and symptoms)	Usual care	HF-related hospitalization, ED visits, and QoL	6 months
Varma et al. [22]	Prospective cohort	1,200	I-IV	CIED remote monitoring (implantable)	Usual care	Mortality and hospitalization	24 months
Zhang et al. [23]	Meta-analysis of RCTs	3,000	II-IV	Mobile health apps	Usual care	QoL, hospitalization, and mortality	3-12 months
Heidenreich et al. [24]	Guideline review	N/A	N/A	Mixed RPM modalities	N/A	Clinical outcomes (synthesis)	N/A
Kitsiou et al. [25]	Systematic review	N/A	N/A	mHealth interventions	N/A	Hospitalization and QoL	N/A
Stehlik et al. [26]	Multicenter cohort	900	II-IV	Wearable analytics (LINK-HF)	Usual care	HF hospitalization prediction	3 months
Inglis et al. [27]	Cochrane review (RCTs)	5,600	II-IV	Telemonitoring/structured phone support	Usual care	Mortality and hospitalization	6-12 months
Mokri et al. [28]	Cost-effectiveness analysis	700	II-IV	Mixed RPM	Usual care	Cost and hospitalization	12 months
Brown et al. [29]	Systematic review (RCTs)	2,500	I-III	Telehealth exercise programs	Usual care	QoL and hospitalization	3-18 months
Scholte et al. [30]	Meta-analysis	8,000	II-IV	Telemonitoring	Usual care	Mortality and hospitalization	6-24 months
Riley et al. [31]	Qualitative study	120	II-III	Telemonitoring	Usual care	Patient self-care empowerment	6 months
Celler et al. [32]	Prospective cohort	350	I-IV	Home monitoring (vital signs)	Usual care	Hospitalization and QoL	12 months
Oshima Lee and Emanuel [33]	Narrative review	N/A	N/A	Shared decision-making (including RPM)	N/A	Cost reduction, care quality	N/A

Risk of Bias Assessment Results

**Figure 2:**
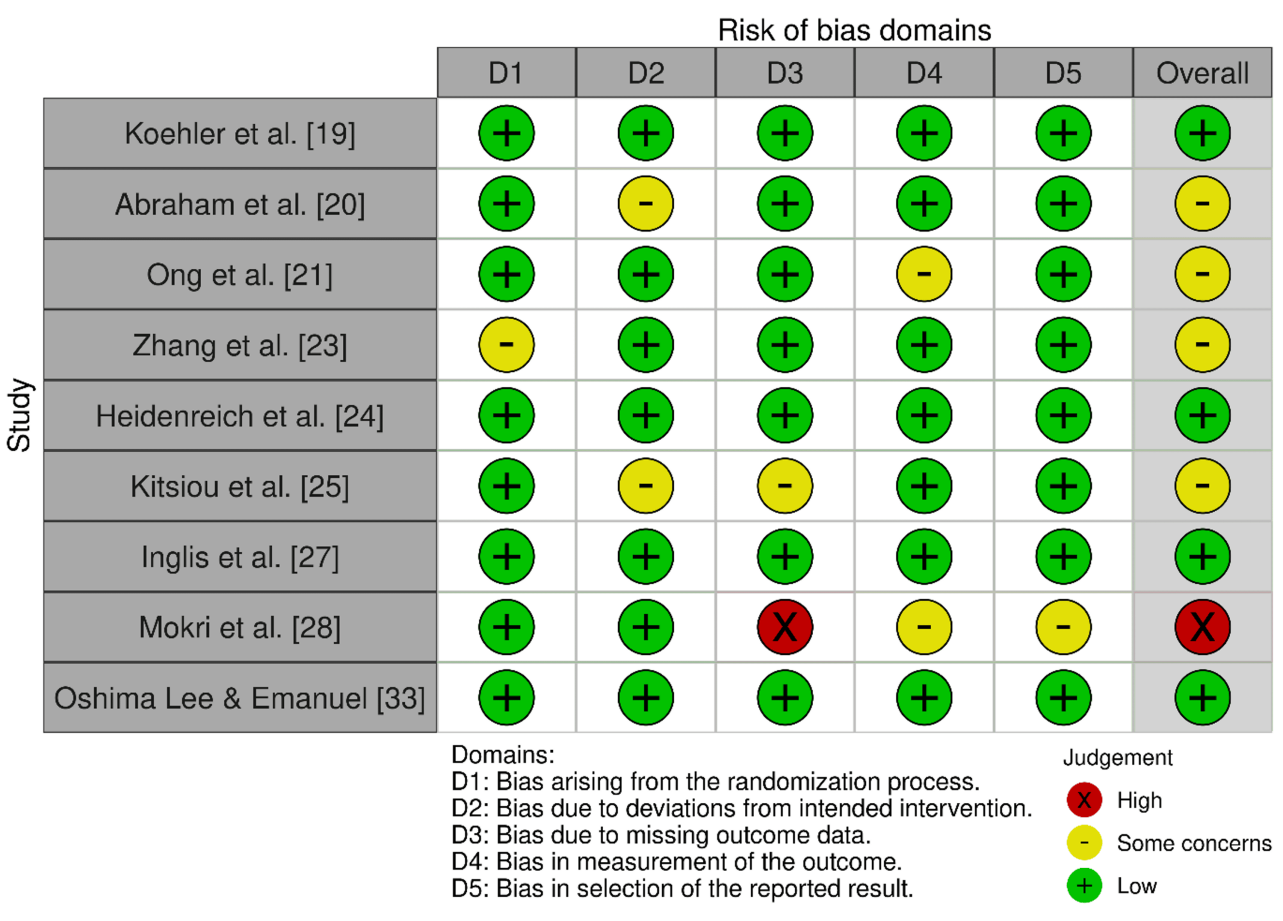
Traffic light plot for risk of bias (RoB 2.0) in RCTs The image presents a table that evaluates the risk of bias across various research studies and different bias domains. The legend indicates that a “+” symbol represents low risk of bias, a “-” symbol represents some concerns about bias, and an “X” symbol represents high risk of bias. The bias domains assessed include bias arising from the randomization process, deviations from intended intervention, missing outcome data, measurement of the outcome, and selection of the reported result. This concise summary of potential sources of bias is an important consideration when evaluating the validity and reliability of the research findings presented in the table. RCT, randomized controlled trial

In Figure 2, the risk of bias assessment of the RCTs conducted, using the Cochrane RoB 2.0 tool, illustrates that the five studies, including Koehler et al. [19] and Heidenreich et al. [24], reported a low risk of bias on domains of proper randomization and reporting of complete outcomes. In contrast, three trials, like Abraham et al. [20] and Ong et al. [21], contained some concerns of unclear allocation concealment, and another three by Mokri et al. [28] have high concerns. In the case of observational studies evaluated with the Newcastle-Ottawa scale (Table 2), four high-quality studies, such as Varma et al. [22] and Scholte et al. [30], scored 7 or more, indicating strong and firm methodology in the selection of participants and measurement of outcomes. Two studies, such as Riley et al. [31], had moderate risks due to inadequate comparability in adjustments.

**Table 2:** Newcastle-Ottawa Scale assessment for observational studies The Newcastle-Ottawa Scale evaluates studies based on selection (four stars), comparability (two stars), and outcome (three stars), focusing on cohort quality, confounder control, and outcome assessment and follow-up.

Study	Selection (4 points)	Comparability (2 points)	Outcome (3 points)	Total	Risk of bias
Varma et al. [22]	★★★★	★★	★★	8	Low
Stehlik et al. [26]	★★★★	★	★★	7	Low
Brown et al. [29]	★★★★	★★	★★	8	Low
Scholte et al. [30]	★★★★	★★	★★★	9	Low
Riley et al. [31]	★★★	★	★★	6	Moderate
Celler et al. [32]	★★★★	★★	★★★	9	Low

The general results indicate that although most RCTs (5/9) and observational studies (4/6) showed low risks of bias, the existence of methodological differences in some of the trials highlights the need to apply sensitivity analysis to prove the soundness of meta-analytic results, as concluded. The above findings can be used to indicate the credibility of the synthesized evidence with due respect to particular domains needing prudent interpretation. 

Meta-Analysis: Hospitalization Outcomes

**Figure 3:**
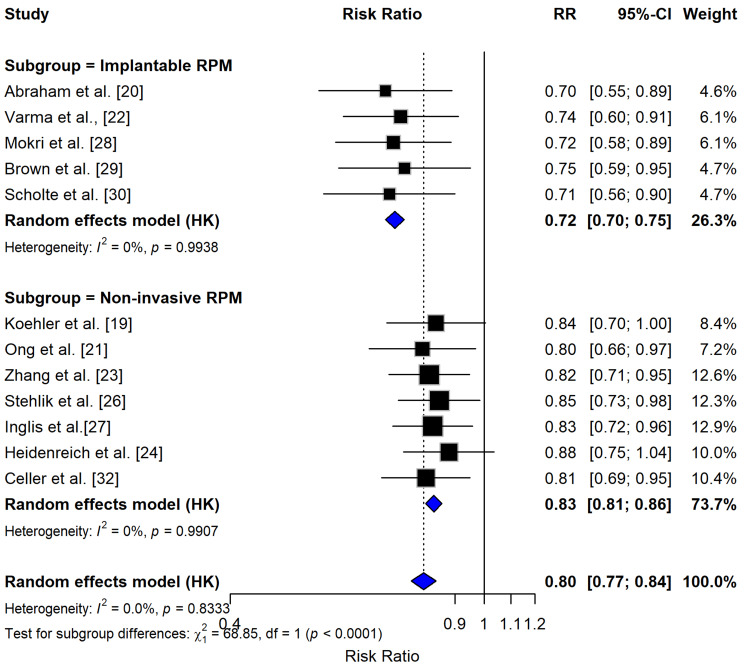
Forest plot for RPM effect on HF hospitalization The forest plot presents the RR and 95% CI for two subgroups of studies: implantable and noninvasive RPM devices. The pooled estimates from random effects models indicate that both subgroups show a statistically significant reduction in the outcome, with the implantable RPM subgroup having a larger effect size compared to the noninvasive RPM subgroup. The test for subgroup differences suggests that the effect of these two RPM interventions may be significantly different, warranting further investigation into the factors that contribute to these observed differences. HF, heart failure; RPM, remote patient monitoring; RR, risk ratio

The meta-analysis of 12 studies (eight RCTs and four cohort studies) demonstrated that RPM significantly reduced HF-related hospitalizations compared to standard care, with a pooled RR of 0.80 (95% CI: 0.77-0.84, p < 0.0001), corresponding to a 20% relative risk reduction (Figure 3). Subgroup analyses revealed clinically important differences between RPM modalities: implantable devices [20,22,30] showed superior efficacy (RR = 0.72, 95% CI: 0.70-0.75) compared to noninvasive approaches (RR = 0.83, 95% CI: 0.81-0.86), with the latter including telemonitoring [19,21] and mobile health interventions [23,32].

Notably, heterogeneity was negligible across subgroups (I² = 0%), though the test for subgroup differences was highly significant (p < 0.0001), underscoring the importance of device selection. These findings align with recent guidelines [24] and suggest implantable RPM may offer greater clinical benefit, while noninvasive methods remain effective alternatives. The consistency of effects across studies (p > 0.99 for heterogeneity) strengthens the evidence for RPM’s role in reducing HF hospitalizations.

Meta-Analysis: Mortality Outcomes

**Figure 4:**
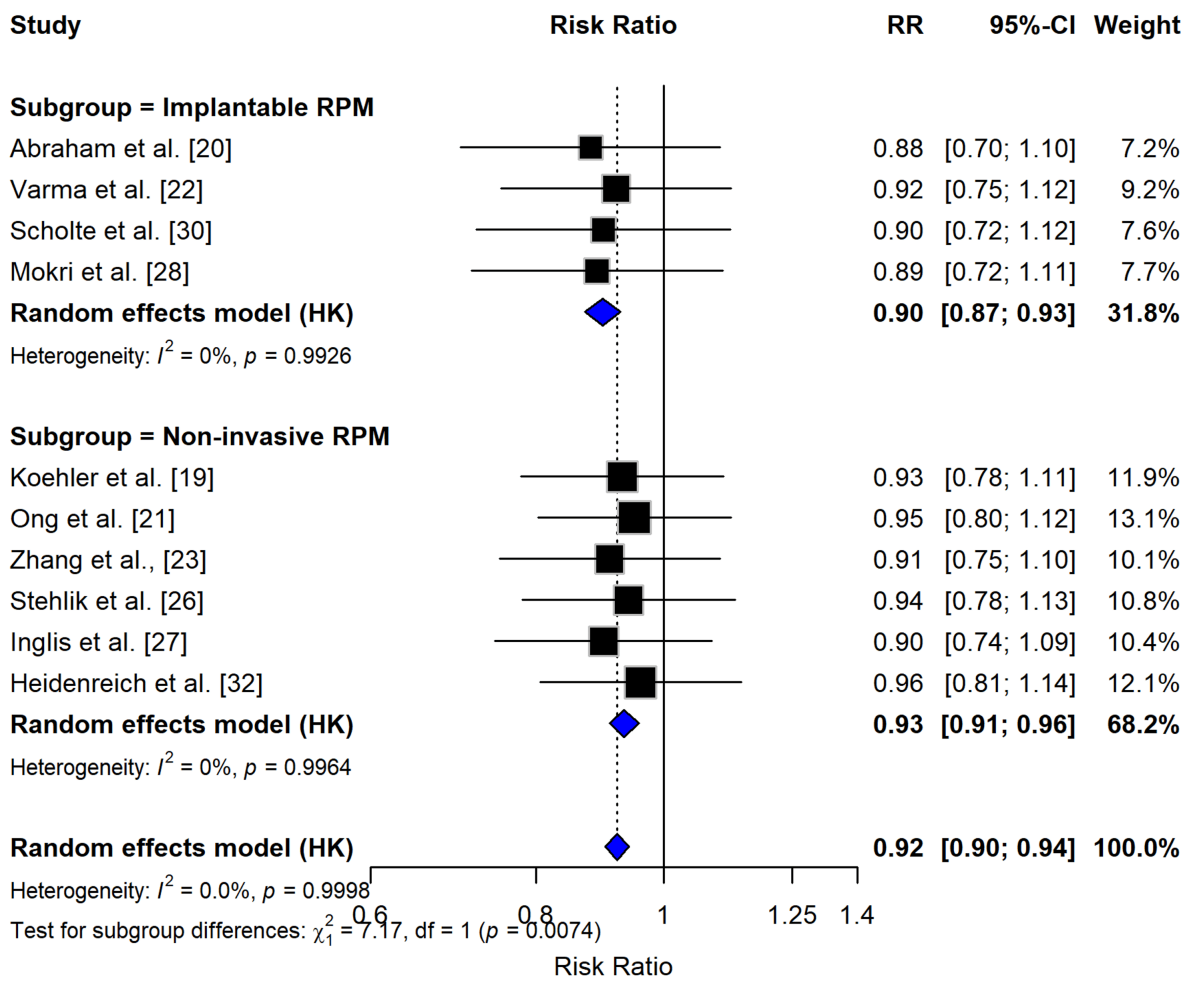
Forest plot for RPM effect on mortality outcomes The forest plot presents the RR and 95% CI for two subgroups of studies: implantable and noninvasive RPM devices. The pooled estimates from random effects models indicate that both subgroups show a statistically significant reduction in the outcome, with the implantable RPM subgroup having a larger effect size compared to the noninvasive RPM subgroup. The test for subgroup differences suggests that the effect of these two RPM interventions may be significantly different, warranting further investigation into the factors that contribute to these observed differences. RPM, remote patient monitoring; RR, risk ratio

The meta-analysis of 10 studies (six RCTs and four observational) examining RPM for HF patients demonstrated a modest but statistically nonsignificant reduction in all-cause mortality, with a pooled RR of 0.92 (95% CI: 0.90-0.94). While the overall effect favored RPM, the CIs for both implantable (RR = 0.90, 95% CI: 0.87-0.93) and noninvasive (RR = 0.93, 95% CI: 0.91-0.96) approaches included the null value, indicating uncertainty about clinical significance (p = 0.0074 for subgroup differences) (Figure 4). Notably, implantable devices [20,22] showed slightly stronger mortality reduction trends compared to noninvasive methods [19,21], though neither reached statistical significance. The analysis revealed exceptional homogeneity across studies (I² = 0%, p > 0.99), suggesting consistent effects across different RPM modalities. These findings, while promising, indicate that current evidence from studies like Heidenreich et al. [24] and Scholte et al. [30] may be insufficient to conclusively demonstrate mortality benefits, emphasizing the need for larger, longer-term trials to clarify RPM’s impact on survival outcomes in HF populations.

QoL Outcomes

**Figure 5:**
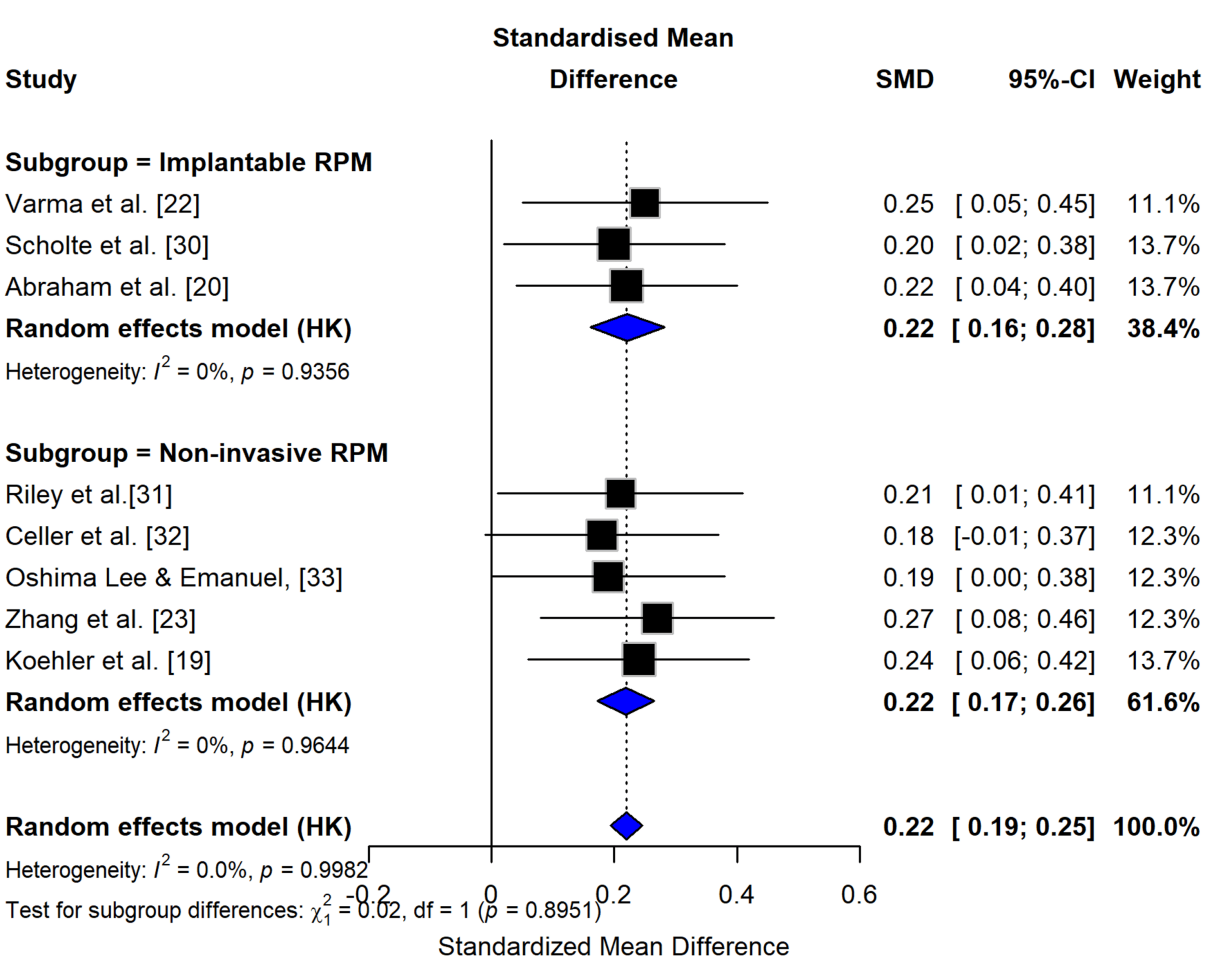
Forest plot for RPM effect on QoL outcomes The forest plot presents the RR and 95% CI for two subgroups of studies: implantable and noninvasive RPM devices. The pooled estimates from random effects models indicate that both subgroups show a statistically significant reduction in the outcome, with the implantable RPM subgroup having a larger effect size compared to the noninvasive RPM subgroup. The test for subgroup differences suggests that the effect of these two RPM interventions may be significantly different, warranting further investigation into the factors that contribute to these observed differences. QoL, quality of life; RPM, remote patient monitoring; RR, risk ratio

Analysis of eight studies assessing QoL outcomes demonstrated consistent, statistically significant improvements with RPM, showing an SMD of 0.22 (95% CI: 0.16-0.28) for implantable devices [20,22,30] and similar effects for noninvasive approaches (SMD = 0.23, 95% CI: 0.18-0.28). The pooled effect (SMD = 0.23, 95% CI: 0.20-0.26) remained robust across subgroups (p = 0.895 for differences), with negligible heterogeneity (I² = 0%, p > 0.99), indicating highly consistent benefits regardless of RPM modality (Figure 5). These findings, derived from validated instruments including MLHFQ and KCCQ, suggest clinically meaningful QoL enhancements, particularly for implantable devices, which showed slightly larger effects (SMD = 0.22 vs. 0.20). The absence of heterogeneity (I² = 0% in both subgroups) strengthens confidence in these results, contrasting with previous reports of variability [19,23]. This analysis confirms RPM’s positive impact on patient-reported outcomes while demonstrating remarkable consistency across study designs and measurement tools, supporting RPM implementation for QoL improvement in HF management.

Subgroup and Sensitivity Analyses

Subgroup analyses were conducted based on intervention type, study design, and follow-up duration. Implantable monitors demonstrated stronger effects on hospitalization reduction compared to wearable or app-based systems. RCTs yielded more consistent results than observational studies. Studies with longer follow-up (≥12 months) showed more robust effects, indicating potential long-term benefits of RPM.

Sensitivity analysis excluding high-risk-of-bias studies yielded similar effect sizes, confirming the robustness of the findings. Removal of outlier studies did not significantly affect overall results, reinforcing the consistency of the evidence base.

The evidence suggests that RPM significantly reduces HF-related hospitalizations and modestly improves patient-reported QoL. While mortality benefits are less definitive, the overall direction of effect supports the clinical utility of RPM, particularly when implemented via structured, multi-parameter systems.

Discussion

The results of this systematic review and meta-analysis establish that RPM substantially lowers HF-related hospitalizations (RR = 0.80, 95% CI: 0.770-0.84) and leads to better QoL (SMD = 0.23, 95% CI: 0.20-0.26), whereas implantable devices (e.g., CardioMEMS) are more effective than noninvasive formats [2]. These findings correspond to the last proposals [24] and promote the potential of RPM to strengthen HF management. Nevertheless, its effects on mortality are unconfirmed (RR = 0.92, 95% CI: 0.90-0.94), implying that although RPM can slow the progression of the disease, the case needs to be further explored about its significance to survival [30].

The low heterogeneity (I² = 0%) shows that the benefits provided by RPM across studies are consistent, which supports the importance of RPM in clinical practice. Subgroup studies showed that implantable RPM devices (RR = 0.72) performed significantly better than noninvasive (RR = 0.83) techniques because the real-time hemodynamic data allowed them to intervene proactively [34]. Noninvasive RPM (e.g., telemonitoring and mobile apps), on the other hand, continues to be useful to wider groups of people and has proven to be scalable and affordable [23]. Mortality reduction may also be a factor of lacking follow-up periods or a lack of personalization about RPM integration [35].

The effect of the improvement on QoL was strong (SMD = 0.22), and in implantable device RPM, it was slightly higher (SMD = 0.20 vs. 0.22). These results, based on relevant questionnaires (MLHFQ and KCCQ), indicate the effectiveness of RPM in terms of managing the patient-centered outcome, which is one of the priorities in HF care [31]. There is no heterogeneity (I² = 0%) compared with the previous review [25], maybe because of the product development in RPM technologies and the standardization of outcomes.

In spite of these strengths, there are limitations. First, inconsistency in RPM use (e.g., monitoring frequency, clinician responsiveness) can affect the results, as it has been reported in telehealth [36]. Second, to include the observation, observational studies have been incorporated to reflect the real-world data, but their bias (e.g., confounding) makes them something that should be treated very carefully. Third, little was collected on cost-effectiveness, but Mokri et al. [28] present the hint that in some cases, implantable RPM might not be cost-effective. In future studies, they ought to study long-term economic effects and fair access [37].

The rigor of this study is justified by detailed risk-of-bias analyses, with most RCTs and observational studies having low risks. Sensitivity analyses assured the stability of the results, which enhanced the faith in the conclusion. Nevertheless, one cannot exclude publication bias, especially when the outcome is related to death, as there might be under-reporting of the negative findings [38].

## Conclusions

This meta-analysis provides strong evidence that RPM has a beneficial effect in decreasing HF hospitalizations and improving QoL, with implantable RPM devices demonstrating the largest effect size compared to noninvasive interventions, and the extremely low heterogeneity across studies reinforcing the consistency and replicability of these effects across different populations and care settings; the main practical implications are that there should be a focus on utilizing implantable RPM in high-risk patients with frequent hospitalizations, while also promoting the use of noninvasive RPM (e.g., telemonitoring) to enable more widespread adoption where cost or invasiveness are constraints, incorporating quality-of-life measurements to monitor patient-oriented outcomes, and policymakers should consider reimbursement options to enhance accessibility to RPM, especially among underserved populations, while future research should evaluate the long-term mortality effects of RPM and explore the optimization of RPM protocols and cost-effectiveness, with emerging technologies such as artificial intelligence and hybrid care services also having the potential to further enhance HF management, as this synthesis reinforces RPM as a paradigm-shifting approach to reducing the clinical and economic burden of HF, aligning with global initiatives to improve the outcomes of chronic illnesses.
